# Recent Advances in Brain Signal Analysis: Methods and Applications 2018

**DOI:** 10.1155/2018/5971086

**Published:** 2018-12-09

**Authors:** Victor Hugo C. de Albuquerque, Plácido Rogerio Pinheiro, Roshan J. Martis, João Manuel R. S. Tavares

**Affiliations:** ^1^Programa de Pós-Graduação em Informática Aplicada, Universidade de Fortaleza, Fortaleza, Brazil; ^2^Department of Electronics and Communication Engineering, Vivekananda College of Engineering and Technology, Puttur, India; ^3^Instituto de Ciência e Inovação em Engenharia Mecânica e Engenharia Industrial, Departamento de Engenharia Mecânica, Faculdade de Engenharia, Universidade do Porto, Porto, Portugal

Signal processing and analysis has been extensively used in the field of neuroscience. For example, (semi)automatic brain-based systems have been increasingly used in various medical applications such as in disease prevention, detection, and diagnosis, rehabilitation, smart education environments, security and authentication, and biometry. These systems are suggested in the literature as accurate, fast, complementary, and alternative tools to aid specialists in their decision-making, by facilitating the analysis and interpretation of brain signals, and to reduce and/or eliminate errors.

The main objective of this special issue was to promote a discussion on the recent advances related to brain signal analysis, both in terms of novel methods and applications, in order to identify potential contributions to the field of neuroscience. This special issue of the *Computational Intelligence and Neuroscience *journal contains 13 original works selected from the 28 submitted. Hence, the selected studies address new trends in methods and techniques applied to different neuroscience applications.

In the first article, Á. Tepper et al. analyzed different power spectrum density methods with the aim to select one that minimizes the calculation time to be used in real time during deep brain stimulation surgery, concluding that the optimum method to perform the real-time spectral estimation of the beta band from the microelectrode recording signal is Welch with Hamming windows of 1.5 seconds and 50% overlap.

The second article, by J. Zhang et al., proposes a semisupervised learning sparse representation classifier with an average coefficient method that performs the classification using the average coefficient of each class, instead of the reconstruction error, and selectively updates the training dataset using new testing data, to improve the performance of the sparse representation classifier. The proposed classifier achieved a better performance than the other three semisupervised learning methods adopted for the comparison purpose.

The third article, by M. A. Porta-Garcia et al., proposes a framework for characterization of phase synchrony relationships between multivariate neural time series applied in EEG signals from P300 speller sessions of four subjects, relying on a proposed clustering algorithm, termed “multivariate time series clustering by phase synchrony.” The proposed solution is able to observe dynamics of phase changes and interactions among channels and can be applied to analyze other cognitive states rather than ERP vs. no event-related potential.

In the fourth article, J. W. M. de Souza et al. propose an approach to measure the similarity between the exam template and the handwritten trace of a patient following the exam template. The proposed solution uses the structural cooccurrence matrix to calculate how close the handwritten trace of the patient is to the exam template and is combined with Naïve Bayes, OPF, and SVM classifiers, showing to be a promising tool for the diagnosis of Parkinson's disease.

The fifth article, by J. Yoon et al., explored the performance of a convolutional neural network on event-related potential (ERP) data to identify the key features that distinguish illiterates of the ERP speller system, in which the P700 peak may be the key feature of ERP as it appears in both illiterate and nonilliterate subjects.

In the sixth article, R. Munoz et al. describe a new method by means of metaheuristics based on the black hole algorithm to improve EEG-based emotion recognition, using the MAHNOB HCI Tagging Database, showing that the black hole algorithm used to optimize the feature vector of the support vector machine classifier obtained an accuracy of 92.56% over 30 executions.

The seventh article, by E. Modica et al., investigates cerebral and emotional reactions during the interaction with food products by EEG and autonomic activities, as caused by the cross-sensory interaction across a number of different products. The study has the obvious limitation of the number of the food packaging products tested, although the sample size of participants was sufficient to reveal significant statistical effects.

In the eighth article, I. Xygonakis et al. propose a multiclass brain-computer interfaces decoding algorithm that uses EEG source imaging, a technique that maps scalp potentials to cortical activations, in order to compensate for low spatial resolution of EEG. The authors obtained a mean accuracy increase of 5.6 % with respect to the conventional application method of a common spatial pattern on sensors.

The ninth article by E. Modica et al. evaluated the cerebral and emotional reaction to the exposure to selected antismoking public service announcements images in an adult sample, which have already been shown to be effective and ineffective for the promotion of an antismoking behaviour.

The tenth article, by H. Jin et al., proposes a method to identify motion intention of different walking states under normal environment, by using the functional near-infrared spectroscopy technology in twenty-two healthy subjects to walk with three different gaits (small step with low speed, small step with mid-speed, and mid-step with low speed), presenting a mean recognition rate of 78.79%.

In the eleventh article, S. Guan et al. describe a novel scheme that combines amplitude-frequency information of intrinsic mode function with a common spatial pattern, namely, AF-CSP, to extract motor imagery features and improve the classification performance. The second-generation nondominated sorting evolutionary algorithm is used to tune hyperparameters for linear and nonlinear kernel one versus one twin support vector machine. The proposed method is promising to achieve higher accuracy in brain-computer interface systems.

The last article of the special issue, by C.-H. Kuo et al., proposes a novel and practical P300-based hybrid stimulus-on-device brain-computer interface architecture for wireless networking applications. The authors showed that, for five subjects, the performance of classification and information transfer rate were improved after calibrations, for instance, 86.00% and 24.2 bits/min before calibrations and 90.25% and 27.9 bits/min after calibrations.

